# Comparative transcriptome analysis reveals significant metabolic alterations in eri-silkworm (*Samia cynthia ricini*) haemolymph in response to 1-deoxynojirimycin

**DOI:** 10.1371/journal.pone.0191080

**Published:** 2018-01-11

**Authors:** Shang-Zhi Zhang, Hai-Zhong Yu, Ming-Jie Deng, Yan Ma, Dong-Qiong Fei, Jie Wang, Zhen Li, Yan Meng, Jia-Ping Xu

**Affiliations:** 1 School of Life Sciences, Anhui Agricultural University, Hefei, Anhui, People’s Republic of China; 2 Analytical and Testing Center of Wenzhou Medical University, Wenzhou, Zhejiang, People’s Republic of China; Institute of Plant Physiology and Ecology Shanghai Institutes for Biological Sciences, CHINA

## Abstract

*Samia cynthia ricini* (Lepidoptera: Saturniidae) is an important commercial silk-producing insect; however, in contrast to the silkworm, mulberry leaves are toxic to this insect because the leaves contain the component 1-deoxynojirimycin (DNJ). A transcriptomic analysis of eri-silkworm haemolymph was conducted to examine the genes related to different metabolic pathways and to elucidate the molecular mechanism underlying eri-silkworm haemolymph responses to DNJ. Eight hundred sixty-five differentially expressed genes (DEGs) were identified, among which 577 DEGs were up-regulated and 288 DEGs were down-regulated in the 2% DNJ group compared to control (ddH_2_O) after 12h. Based on the results of the functional analysis, these DEGs were associated with ribosomes, glycolysis, N-glycan biosynthesis, and oxidative phosphorylation. In particular, according to the KEGG analysis, 138 DEGs were involved in energy metabolism, glycometabolism and lipid metabolism, and the changes in the expression of nine DEGs were confirmed by reverse transcription quantitative PCR (RT-qPCR). Thus, DNJ induced significant metabolic alterations in eri-silkworm haemolymph. These results will lay the foundation for research into the toxic effects of DNJ on eri-silkworm as a model and provide a reference for the exploitation of new drugs in humans.

## Introduction

Mulberry trees are perennial woody plants that have great economic importance because their leaves are used to feed the silkworm [1]. Mulberry leaves also contain a large number of traditional Chinese herbal medicines. One of the main active compounds in mulberry latex is the natural 1-deoxynojirimycin (DNJ) [2]. DNJ is a D-glucose analogue with promising biological activity *in vivo*, contains an NH group in place of the oxygen atom of the pyranose ring and is also distributed in other plants, including *Hyacinthus orientalis*, *Commelina communis* and *Adenophora triphylla* var. *japonica* [3,4]. Recently, DNJ has been reported to have a significant effect on improving diabetic conditions by inhibiting the activity of α-glucosidase. Therefore, it has gained extensive attention for potential use as a medical food to control postprandial blood glucose levels [5,6]. Additionally, DNJ and its derivatives have also shown potential antiviral activity and the ability to inhibit tumours and hypolipidaemia [7–9].

*Samia cynthia ricini* (Lepidoptera: Saturniidae; Bombycoidea) is a commercial silk-producing insect originating from India, China and Japan [10]. Eri-silkworm are mainly reared on castor leaves rather than mulberry leaves because mulberry leaves contain large amounts of a 56-kDa defence protein designated mulatexin (MLX56) and alkaloids, such as DNJ, in addition to the latex. These active substances are lethal to *S*. *c*.*ricini*, *Mamestra brassicae Linnaeus*, and several other herbivorous insects [11,12]. Eri-silkworm larvae have been applied to detect and assess the level of plant defence against herbivorous animals. Thus, eri-silkworm represents a good pharmacological model for researching the mechanism of action of DNJ [13,14].

Over the past few decades, technological innovations have enabled researchers to construct an overview of the changes at the transcriptional level in insects following xenobiotic stimulation, including insecticides and viruses [15,16]. During the feeding stage, insects store glycogen and triglycerides as energy reserves in the fat body. Trehalose and diglycerides are released from the fat body into the haemolymph to meet the insect’s energy demands [17]. Therefore, we utilized the fifth-instar eri-silkworm haemolymph to research the toxic effects of DNJ using a transcriptomic analysis. As shown in our previous study, DNJ has a positive impact on reverse glycometabolism by modulating glycometabolism and inhibiting glucogenesis and energy metabolism in the fourth-instar eri-silkworm haemolymph [18]. In the eri-silkworm midgut, DNJ not only exerts a potent negative effect on energy metabolism and glycometabolism but also modulates lipid metabolism [19]. However, the metabolic mechanism underlying the fifth-instar eri-silkworm response to DNJ is unclear, particularly at the transcriptional level.

Transcriptomics were applied to examine the differences in transcript levels in *S*. *c*.*ricini* haemolymph in response to DNJ and to reveal the toxic effects of DNJ on *S*. *c*.*ricini* at the transcriptional level. A large number of DEGs were identified that are involved in glycometabolism, lipid metabolism and energy metabolism in eri-silkworm. This study will lay the foundation for a better understanding of the effects of DNJ on glycometabolism, lipid metabolism and energy metabolism and provide a reference for further studies of drug targets using DNJ.

## Materials and methods

### Eri-silkworm rearing conditions and experimental design

*S*. *c*.*ricini* larvae were provided by the Sericultural Research Institute of Chinese Academy of Agricultural Sciences, Zhenjiang. The larvae were reared on fresh castor leaves at 24 ± 1°C with 75% humidity and a 12:12 L:D photoperiod. The newly exuviated fifth-instar larvae were randomly selected, divided into two groups, and then fed 5 μL of 2% DNJ (J&K Chemicals, China) or ddH_2_O. The larval haemolymph was collected from control and treatment groups after 12h. A small amount of thiourea and TRIzol (Invitrogen, Grand Island, NY, USA) was added to the haemolymph and immediately stored at -80°C until further use.

### RNA extraction

Total RNA was extracted from eri-silkworm haemolymph (control and 2% DNJ-treated) using TRIzol (Invitrogen), according to the manufacturer’s protocol. The A_260/A280_ ratios and the concentrations were examined using a Qubit RNA Kit and a Qubit 2.0 Fluorometer (Life Technologies, CA, USA). Ultimately, RNA integrity was assessed using the RNA Nano 6000 Assay Kit and an Agilent 2100 Bioanalyzer (Agilent, Palo Alto, CA, USA) and was confirmed using 1% agarose gel electrophoresis.

### Library preparation and Illumina sequencing

Fragment interruption, cDNA synthesis, the addition of adapters, PCR amplification and Illumina sequencing were performed by Beijing Novogene Bioinformatics Technology Co., Ltd. (Beijing, China). The sequencing libraries were constructed using a NEBNext® Ultra™ RNA Library Prep Kit for Illumina® (NEB, Ipswich, MA, USA), according to the manufacturer’s recommendations, and index codes were added to attribute sequences to each sample. The quality of these libraries was assessed with an Agilent 2100 Bioanalyzer system. Index-coded samples were clustered with a cBot Cluster Generation System using a TruSeq PE Cluster Kitv3-cBot-HS (Illumina, San Diego, CA, USA), according to the manufacturer’s instructions. The libraries were sequenced using an Illumina HiSeq™ 2000 platform, and 100-bp paired-end reads were generated. The fastq format raw reads were processed using in-house Perl scripts. Clean reads were obtained by removing reads containing adapters or poly-N sequences and removing low quality reads from the raw data. Q20 (the percentage of bases with a Phred value > 20), Q30 (the percentage of bases with a Phred value > 30), and the GC (base G and C) content of the clean data were calculated. All downstream analyses were performed based on clean and high quality data.

### Reads assembly and functional annotation

The left files (read 1 files) from all samples were pooled into one large left.fq file. The right files (read 2 files) were pooled into one large right.fq file. Transcriptome assembly was accomplished based on the left.fq and right.fq files using Trinity [20]. The min_kmer_cov was set to 2 and all other parameters were set to default values. Gene function was annotated based on the following databases: Nr (NCBI non-redundant protein sequences), KOG/COG (Clusters of Orthologous Groups of proteins), Swiss-Prot (a manually annotated and reviewed protein sequence database), KO (KEGG Orthologue database), and GO (Gene Ontology). All searches were performed with an *E*-value < 10^−5^. Fragments per kilobase of transcript per million fragments mapped (FPKM) were calculated to represent the expression level of the unigenes.

### Identification and analysis of DEGs

DEGs were identified using the DESeq R package (1.10.1). DESeq includes statistical routines for determining DEGs based on the negative binomial distribution. The resulting P-values were adjusted using the Benjamini and Hochberg approach for controlling the false discovery rate. Genes were designated as differentially expressed when the adjusted *p*-value was < 0.05 and |log_2_ (fold change)| > 0. GO and Kyoto Encyclopedia of Genes and Genomes (KEGG) enrichment analyses were conducted using the GOseq R packages and KOBAS software [21, 22].

### Reverse transcription quantitative PCR (RT-qPCR) analysis

The relative expression levels of 11 randomly selected DEGs were confirmed by RT-qPCR to validate the reliability of the transcriptome data. Additionally, 9 genes related to glycometabolism, lipid metabolism and energy metabolism were validated. The primers are listed in S1 Table. Total RNA was extracted from the haemolymph of the control and treatment groups using TRIzol reagent. The concentration of each RNA sample was adjusted to 1 μg/μL with nuclease-free water and total RNA was reverse transcribed in a 20-μL reaction system using the PrimeScript™ RT Reagent Kit with gDNA Eraser (TaKaRa, Dalian, China). RT-qPCR was conducted in a 25-μL reaction mixture containing 12.5 μL of SYBR Premix Ex Taq (TaKaRa). PCR amplification was performed in triplicate wells. *S*.*c*.*ricini β-actin* (*ScActin*) was used as a reference gene. The thermal cycling profile consisted of an initial denaturation step at 95°C for 30 s and 40 cycles of 95°C for 5 s and 60°C for 30 s. The reactions were performed in 96-well plates with a Multicolor Real-time PCR Detection System (Bio-Rad, Hercules, CA, USA). Relative expression levels were calculated using the 2^−ΔΔCt^ method according to a previously reported protocol [23]. Three biological replicates were performed for each sample, and each biological replicate included three technical replicates. The statistical analysis was conducted using ANOVA and the LSD *post hoc* test using SPSS (p < 0.01).

## Results

### Illumina sequencing, reads assembly and functional annotation

**Fig 1:**
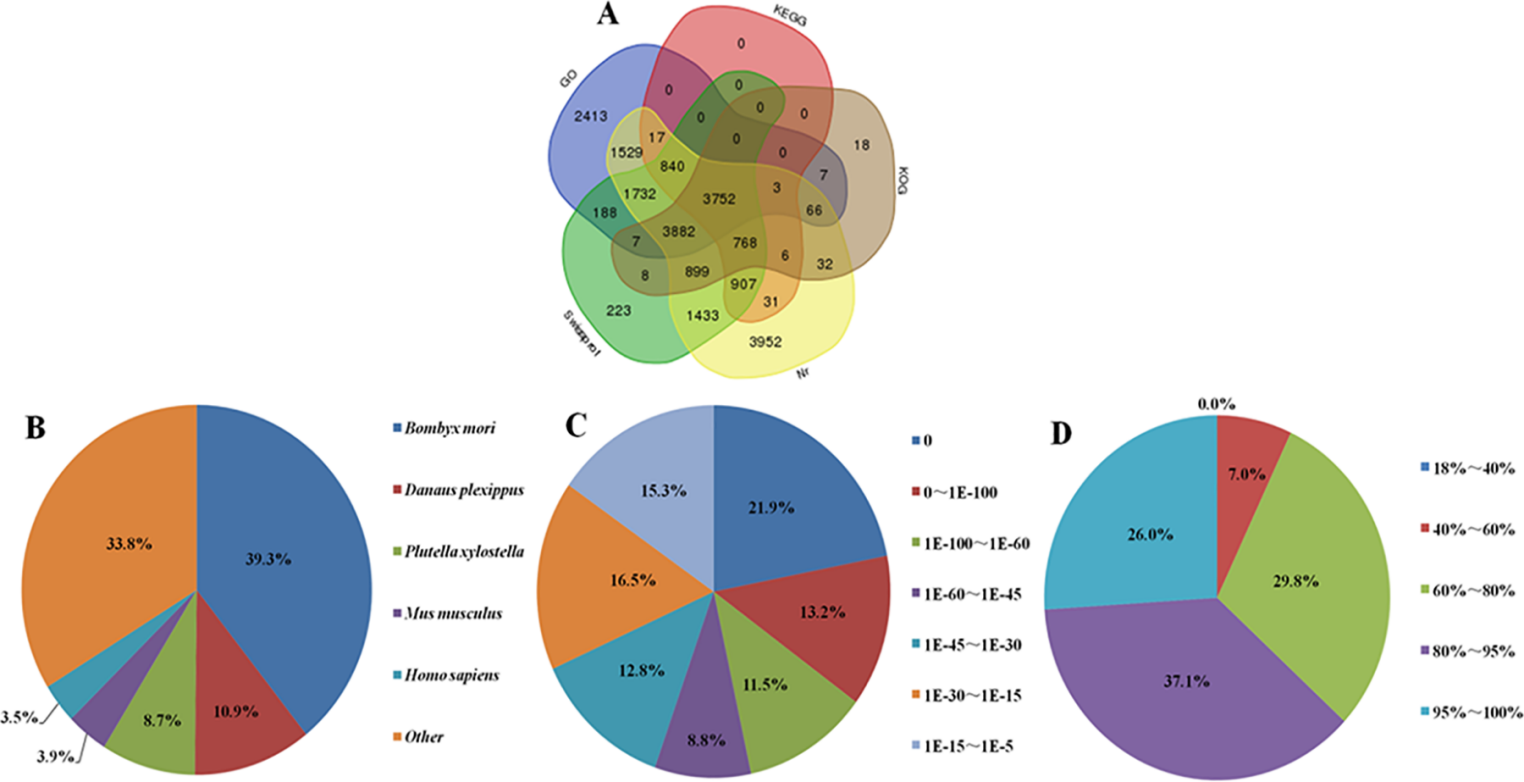
Characteristics of the homology search of Illumina sequences against the Nr database. (A) Venn diagram of unigenes annotated by Blastx against five protein databases. (B) The species distribution is shown as a percentage of the total homologous sequences with an *E*-value of at least 1.0E^-5^. (C) *E*-value distribution of BLAST hits for each unique sequence with a cut-off *E*-value 1.0E^-5^. (D) Similarity distribution of the top BLAST hits for each sequence.

RNA samples from the haemolymph of the two groups (2% DNJ-treated and ddH_2_O-treated) were sequenced on an Illumina HiSeq™ 2000 platform; three biological replicates and three technical replicates were included. We generated 46,811,398, 57,836,584, 48,797,240, 44,655,898, 42,290,810 and 42,821,388 raw reads from the 2% DNJ-treated and control groups, respectively. After stringent quality assessment and data filtering using the Trinity *de novo* assembly programme, 45,386,222, 56,099,312, 47,216,414, 43,437,882, 41,263,002, 41,636,180 clean reads were obtained. The Q20 (sequencing error rate < 1%) and Q30 (sequencing error rate < 0.1%) were greater than 96.04% and 90.83%, and the GC contents were 45.8%, 45.72%, 46.42%, 45.23%, 46.43% and 46.65%, respectively (S2 Table). All short-read sequences were assembled into 86,319 transcripts and 73,296 unigenes (S3 Table). Thus, the quality and accuracy of the sequencing data were sufficient for further analysis. Using a blastx programme with a cutoff *E*-value of 10^−5^, 73,296 unigenes were annotated to different protein databases, including the Nr, KEGG, KOG, GO and Swiss-Prot databases (Fig 1A). Based on an analysis of the Venn diagram, 19,846 unigenes had significant matches in the Nr database and 14,639 unigenes had matches in the Swiss-Prot database. Additionally, 14,436, 20,083 and 9,448 unigenes were annotated in GO, KEGG and KOG, respectively. A total of 2,413 unigenes were annotated only in the GO database, 223 unigenes were assigned only in Swiss-Prot and 3,952 and 18 unigenes were annotated only by Nr and KOG, respectively. In addition, 3,752 unigenes were assigned to a homologue in all five databases (Fig 1A). The species, *E*-value and similarity distribution were analysed by evaluating the matched unigenes from the BLASTX results returned from the Nr protein database. Regarding the species distribution, the highest percentage of unigenes was matched to *Bombyx mori* (39.3%), followed by *Danaus plexippus* (10.9%) and *Plutella xylostella* (8.7%) (Fig 1B). The *E*-value distribution of the best hits against the nr database showed that 55.4% of the sequences displayed significant homology (*E*-value < 1.0E^-45^), and the *E*-values of most of the unigenes ranged from 1.0E^-15^ to 1.0E^-45^ (Fig 1C). On the other hand, according to the similarity distribution, 26% of the unigenes exhibited significant homology greater than 95%, and only 7.0% of the sequences displayed homology less than 60% (Fig 1D).

### RT-qPCR validation of differentially expressed transcripts

**Fig 2:**
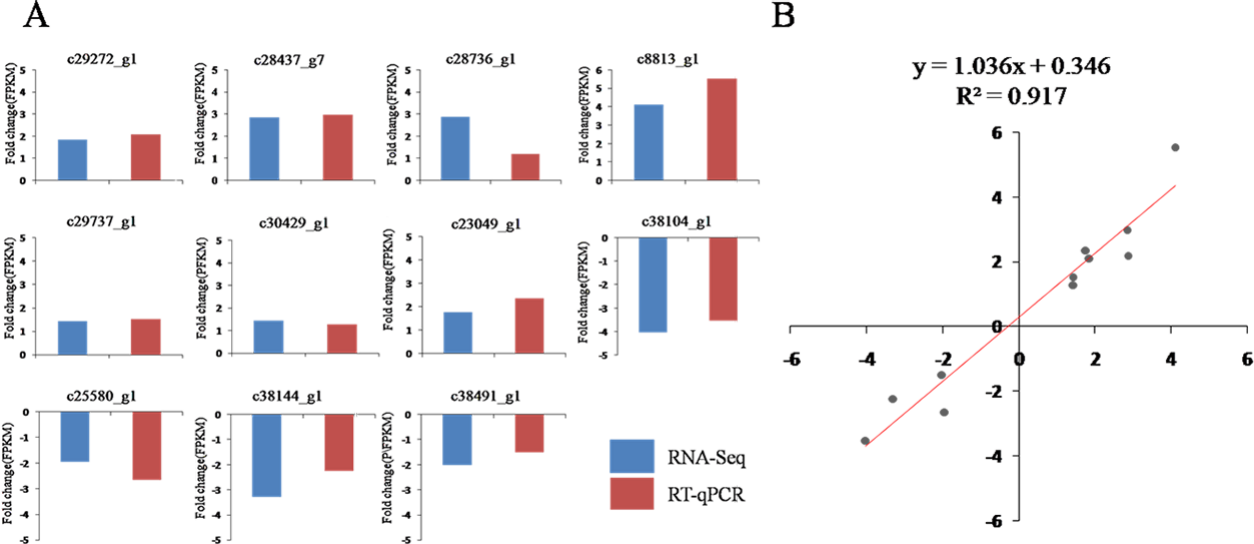
Correlation between gene expression ratios obtained from the transcriptome data and RT-qPCR data. (A) Expression ratios (FPKM fold change) obtained from transcriptome data (blue) and RT-qPCR data (red). (B) Lineage analysis between the transcriptome and RT-qPCR data. The ratios obtained by RT-qPCR (*Y-*axis) were plotted against the ratios obtained by RNA-Seq (*X*-axis).

The relative expression levels of 11 randomly selected genes were analysed by RT-qPCR to validate the reliability of the transcriptome sequencing data (Fig 2A). The trends in the RT-qPCR data were consistent with the transcriptome data. A linear regression analysis of the correlation between RT-qPCR and RNA-Seq data showed an R^2^ (goodness of fit) value of 0.917 and corresponding slope of 1.306 (Fig 2B), suggesting a strong positive correlation between the RT-qPCR and transcriptome data. Therefore, the transcriptome data were suitable for further analysis.

### Identification of DEGs in response to DNJ

**Fig 3:**
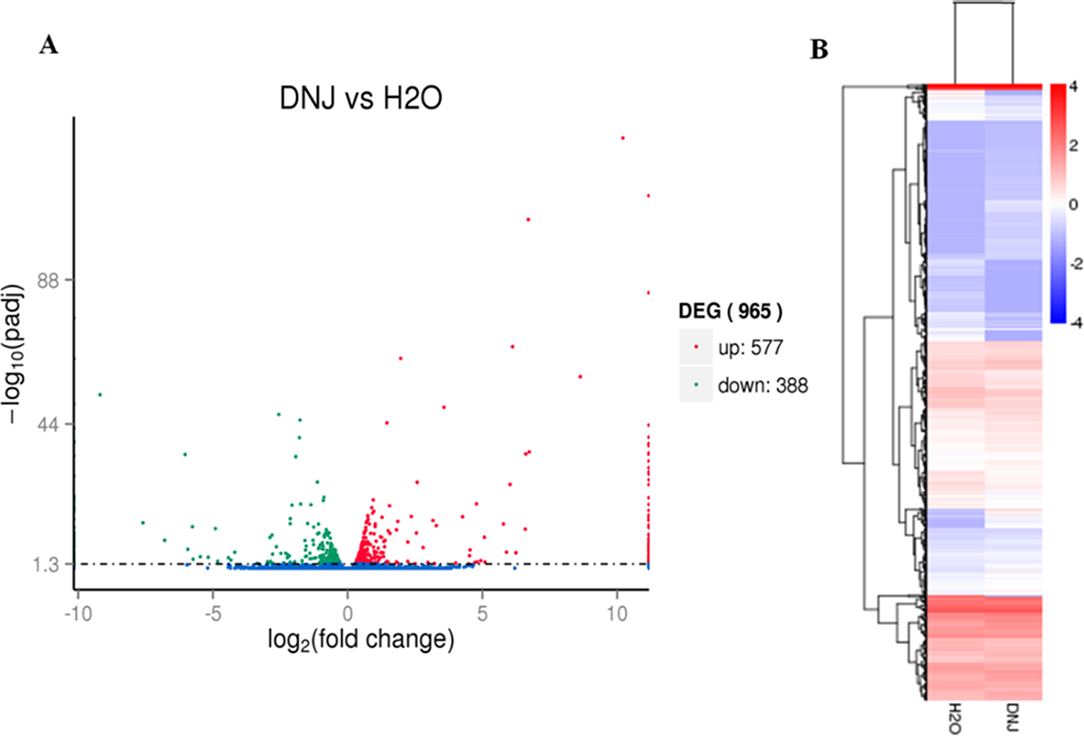
Identification and hierarchical cluster analysis of differentially expressed genes. (A) Scatter diagram for each gene. The blue, red and green points represent no difference in expression, up-regulated and down-regulated unigenes, respectively. (B) Hierarchical clustering of DEGs between the control and 2% DNJ-treated groups. Columns indicate different samples. Rows represent different DEGs. Blue bands indicate a low gene expression level, and red bands indicate a high gene expression level.

In this study, differentially expressed genes between control and treated groups were defined using adjusted *p*-values. Nine hundred fifty-six DEGs were identified in the 2% DNJ-treated group compared with the control group, of which 577 DEGs were up-regulated and 388 DEGs were down-regulated (Fig 3A). We performed hierarchical clustering of all DEGs based on the log10 (RPKM+1) values of the two groups to determine the expression patterns of the identified genes (Fig 3B). DNJ significantly altered the transcriptional profile of the DEGs. The expression of a greater number of genes was up-regulated than down-regulated in the DNJ-treated group compared with the control group.

### Functional annotation of DEGs

**Fig 4:**
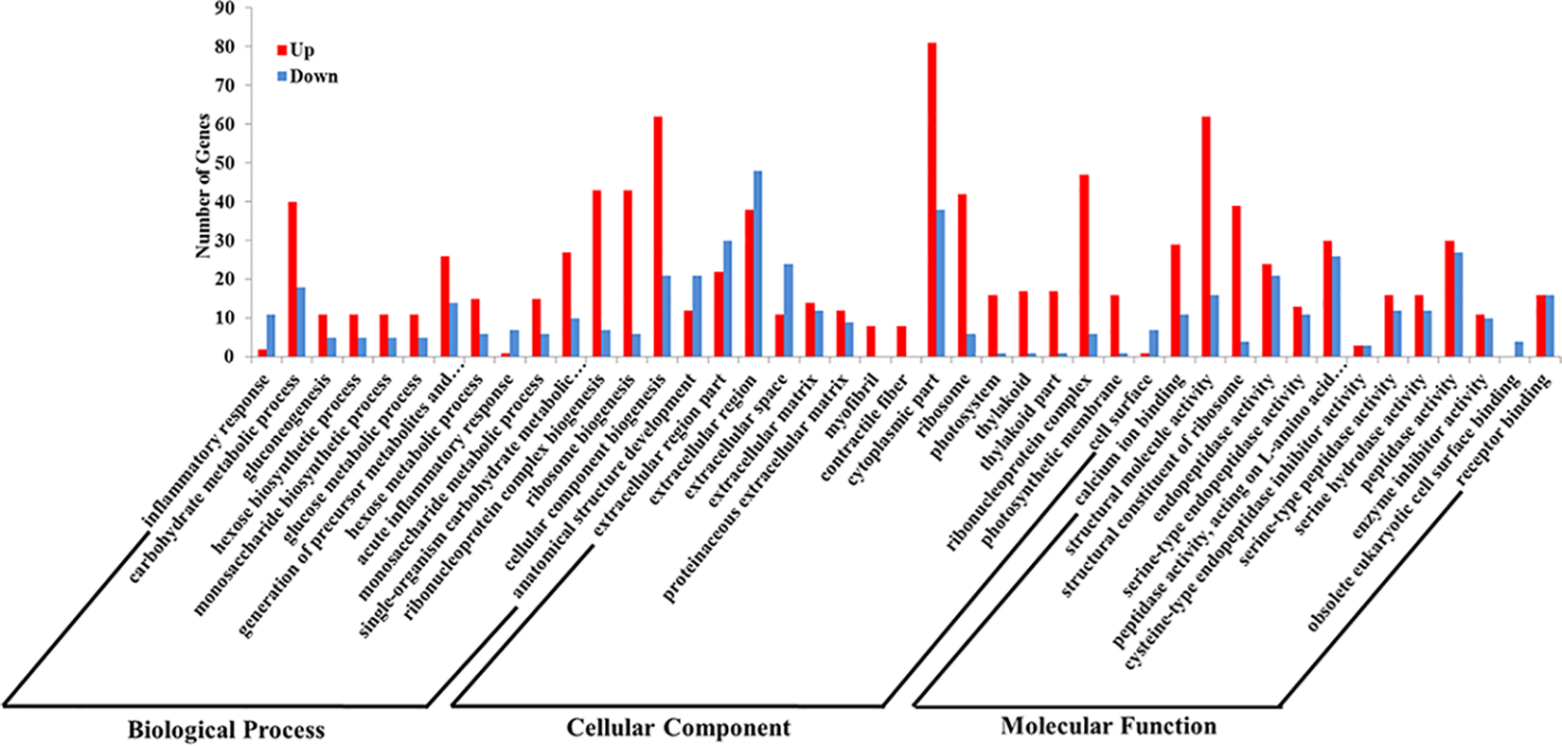
GO categories of the differentially expressed genes (DEGs). The annotated DEGs were classified into the cellular component, molecular function and biological process categories by WEGO according to the GO terms. Red and blue bars indicate up-regulated and down-regulated DEGs, respectively.

GO and KEGG enrichment analyses were performed to further analyse the functions of the DEGs. The GO project provides structured and controlled vocabularies and classifications for the annotation of genes that cover several domains of molecular and cellular biology [24]. Seven hundred eight unigenes were assigned into three main GO categories: cellular component, molecular function and biological process. The GO analysis of the up-regulated and down-regulated DEGs is shown in Fig 4. For the biological process category, the up-regulated genes were mainly involved in cellular component biogenesis, ribosome biogenesis, ribonucleoprotein complex biogenesis and carbohydrate metabolic process, and down-regulated genes were involved in anatomical structure development. For the cellular component category, up-regulated genes were assigned into the cytoplasmic part, followed by the ribonucleoprotein complex, and the down-regulated genes were assigned to the extracellular region. For the molecular function category, the up-regulated genes were mainly related to structural molecule activity, followed by structural constituent of ribosome, and the down-regulated genes were associated with peptidase activity.

**Fig 5:**
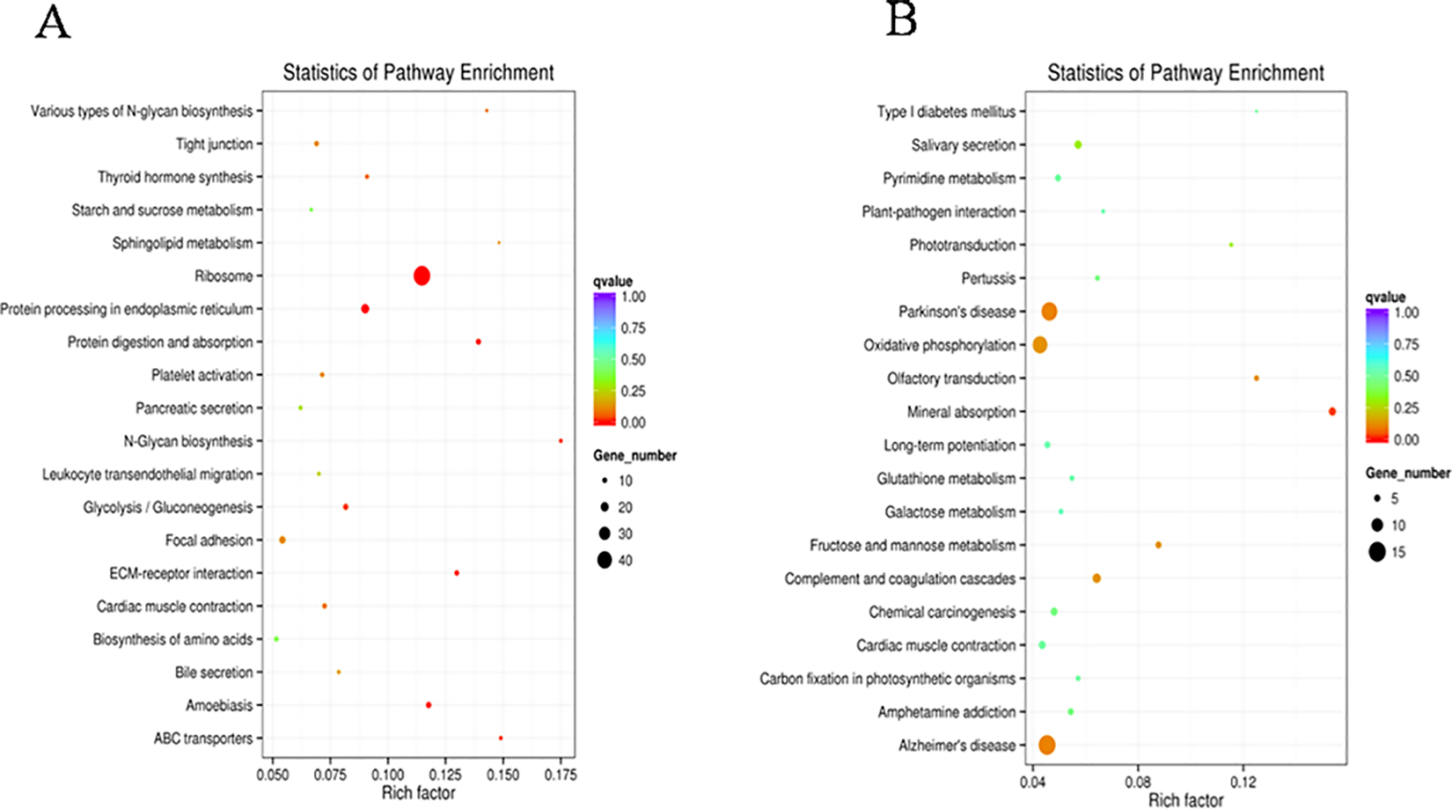
KEGG enrichment analysis of DEGs. Scatter diagram of KEGG pathways. The *X*-axis indicates the enrichment factor. The *Y*-axis indicates different pathways. (A) Up-regulated DGEs. (B) Down-regulated DGEs.

The KEGG pathway analysis provides classifications that are valuable for studying the complex biological functions of genes [25]. According to the KEGG pathway enrichment analysis, the up-regulated DEGs were significantly enriched in six pathways, including ribosomes, the endoplasmic reticulum and protein processing, glycolysis, N-glycan biosynthesis, starch and sucrose metabolism and biosynthesis of amino acids (Fig 5A). The down-regulated DGEs were significantly enriched oxidative phosphorylation and complement and coagulation cascades, fructose and mannose metabolism (Fig 5B).

### Analysis of DEGs related to glycometabolism, lipid metabolism and energy metabolism

**Fig 6:**
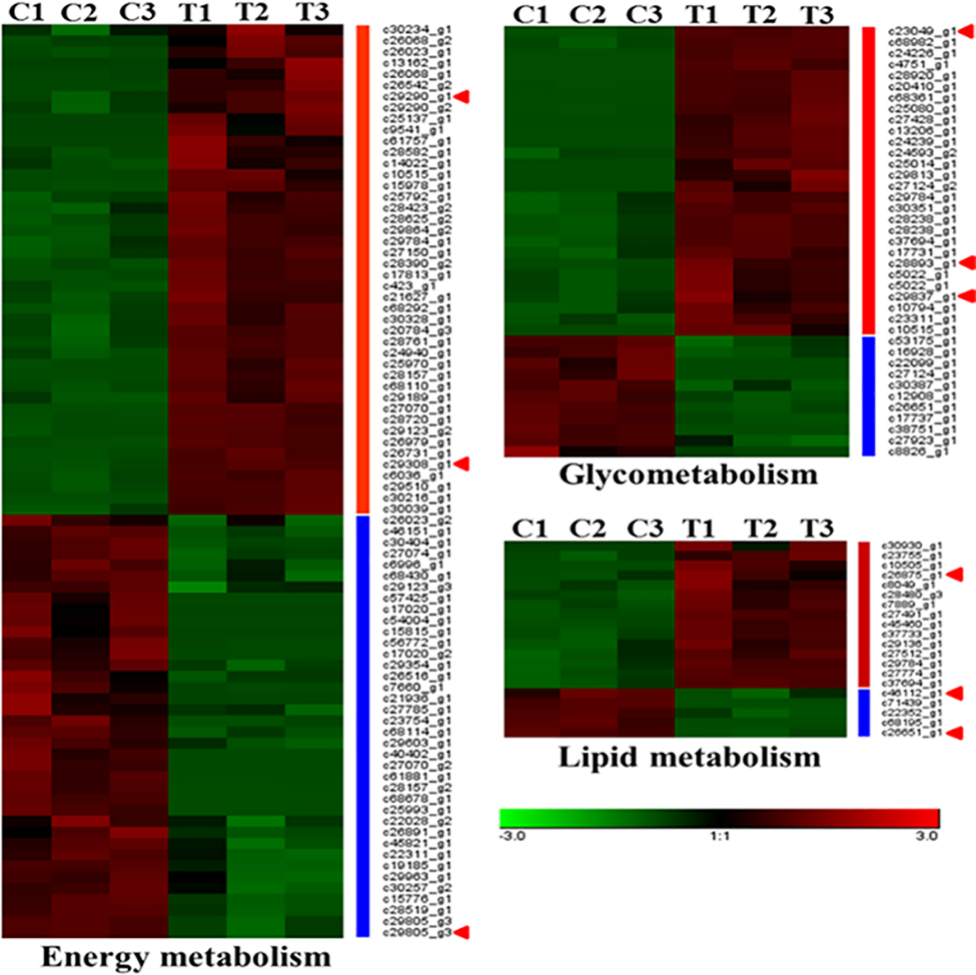
Expression patterns of DEGs related to glycometabolism, lipid metabolism and energy metabolism in the 2% DNJ-treated group and control group. Each row represents a different gene, with green and red indicating low and high levels of gene expression, respectively. C: control, T: 2% DNJ treatment. The numbers 1, 2 and 3 represent the three biological replicates.

**Fig 7:**
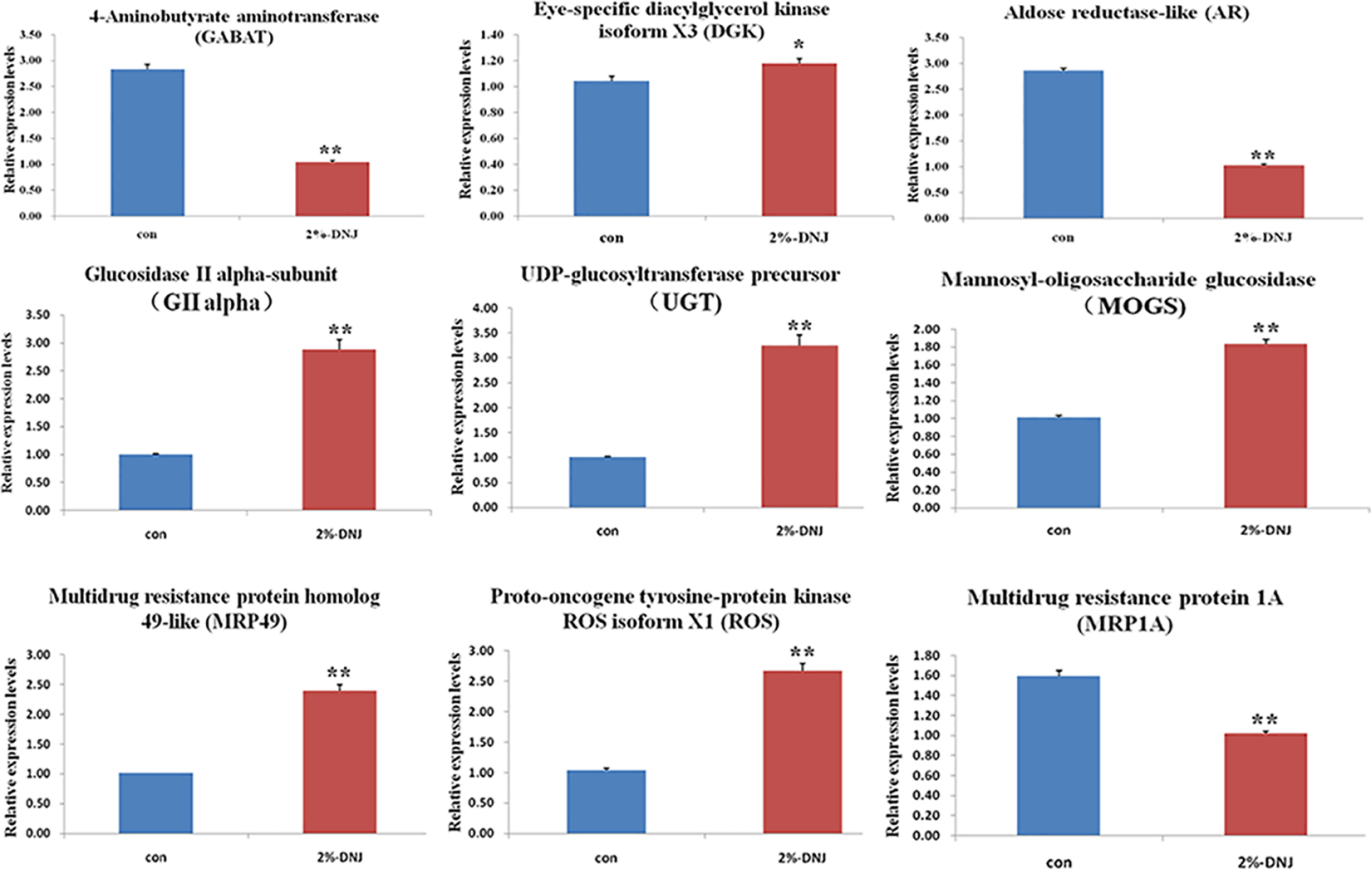
RT-qPCR analysis of the expression patterns of nine genes in eri-silkworm haemolymph. The different colours represent different groups; blue and red represent the control 2% DNJ-treated groups, respectively (* p < 0.05, **p < 0.01).

In this study, based on results of GO annotation and KEGG pathway enrichment analysis, the DEGs were mainly enriched in glycometabolism, lipid metabolism and energy metabolism (Fig 6 and S4 Table). Eighty-one DEGs related to energy metabolism were identified, of which 43 DEGs were up-regulated and 38 were down-regulated in the 2% DNJ-treated group compared with the control. Thirty-seven DEGs related to glycometabolism were identified, of which 26 DEGs were up-regulated and 11 DEGs were down-regulated in the 2% DNJ-treated group compared with the control. Additionally, 20 DEGs were associated with lipid metabolism, of which 15 DEGs were up-regulated and 5 DEGs were down-regulated in the 2% DNJ-treated group compared with the control. Nine genes were selected for further analysis of their expression patterns using RT-qPCR (Table 1 and Fig 7). Three genes were related to lipid metabolism, including *4-aminobutyrate aminotransferase* (*GABAT*), *eye-specific diacylglycerol kinase isoform X3* (*DGK*), and *aldose reductase-like* (*AR*). Based on the RT-qPCR results, *GABAT* and *AR* were down-regulated, and *DGK* was up-regulated in the 2% DNJ-treated group compared with the control. Three genes were associated with glycometabolism, including *UDP-glucosyltransferase precursor* (*UGT*), *mannosyl-oligosaccharide glucosidase* (*MOGS*), and *glucosidase II alpha-subunit* (*GII alpha*). According to the RT-qPCR analysis, these three genes were up-regulated in the 2% DNJ-treated group compared with the control. On the hand, 3 genes were involved in energy metabolism, including *multidrug resistance protein homologue 49-like* (*MRP49*), *proto-oncogene tyrosine-protein kinase ROS isoform X1* (*ROS*), and *multidrug resistance protein 1A* (*MRP1A*). The RT-qPCR analysis indicated that *MRP-49* and *ROS* were up-regulated, and *MRP1A* was down-regulated in the 2% DNJ-treated group compared with the control. These results of the RT-qPCR analysis are consistent with the transcriptome sequencing data. Thus, DNJ caused significant alterations in lipid metabolism, glycometabolism and energy metabolism in eri-silkworm haemolymph.

**Table 1:** Identification of DEGs related to glycometabolism, lipid metabolism and energy metabolism.

Gene ID	Nr annotation	2% DNJ FPKM	H_2_O FPKM	2% DNJ vs. H_2_O ratio	*P*-value
c46112_g1	4-aminobutyrate aminotransferase, mitochondrial (*Bombyx mori*)	1.27	2.25	0.57	0.00018
c26875_g1	eye-specific diacylglycerol kinase isoform X3 (*Bombyx mori*)	3.19	1.82	1.75	1.53E-05
c26651_g1	aldose reductase-like (*Bombyx mori*)	14.36	33.90	0.42	4.59E-11
c29290_g1	multidrug resistance protein homologue 49-like (*Bombyx mori*)	46.33	24.00	1.93	6.46E-06
c29308_g1	proto-oncogene tyrosine-protein kinase ROS isoform X1 (*Bombyx mori*)	2.12	0.69	3.07	8.44E-23
c29805_g3	multidrug resistance protein 1A (*Bombyx mori*)	2.52	5.80	0.43	2.53E-10
c23049_g1	glucosidase II alpha-subunit (*Spodoptera frugiperda*)	105.79	59.97	1.76	2.24E-19
c29737_g1	mannosyl-oligosaccharide glucosidase (*Bombyx mori*)	6.48	4.53	1.43	0.0001
c28893_g1	UDP-glucosyltransferase precursor (*Bombyx mori*)	13.85	6.68	2.07	5.99E-14

## Discussion

As an alkaloid from mulberry, DNJ shows substantial inhibitory activity towards α-glucosidase *in vitro*, which may be beneficial for suppressing abnormally high blood glucose levels [3]. In our previous study, we used eri-silkworm as a model to study the toxic actions of DNJ via metabonomics, and DNJ modulated glycometabolism and inhibit glucogenesis and energy metabolism [18,19]. DNJ is reported to exert a significant inhibitory effect on the activity of α-glucosidase. However, the overall effects of DNJ on glycometabolism, energy metabolism and lipid metabolism in the eri-silkworm are unclear. Moreover, genes involved in glycometabolism, lipid metabolism and energy metabolism have not been researched at the transcriptional level. In the present study, transcriptome sequencing was performed on fifth-instar haemolymph samples from two groups (2% DNJ-treated and control) at 12h after treatment and 73,296 unigenes were obtained (S3 Table). Based on adjusted *p*-values, 965 DEGs were identified. To our surprise, a greater number of up-regulated DEGs (577) was identified than down-regulated DEGs (288) (Fig 3A). DNJ stimulation promoted the up-regulation of gene expression in *S*. *c*.*ricini*. Many xenobiotics also induce the up-regulation of host gene expression. For example, Hou et al. [26] employed transcriptomics to screen the DEGs of silkworm larvae during an early response to *Beauveria bassiana*, and 960 DEGs were up-regulated and 470 DEGs were down-regulated. According to the results of the KEGG pathway enrichment analysis, up-regulated genes were significantly enriched in four pathways, including ribosome, glycolysis/gluconeogenesis, N-glycan biosynthesis and starch and sucrose metabolism (Fig 5A). Down-regulated DEGs were significantly enriched in oxidative phosphorylation and fructose and mannose metabolism (Fig 5B).

### Glycometabolism

Glycometabolism plays an important role in the physiological balance of living organisms [27]. As shown in our previous studies, the glycometabolism pathway was impaired in the haemolymph and midgut of fourth-instar larvae after oral administration of DNJ [18,19]. In this study, based on the KEGG database analyses, the up-regulated genes related to glycometabolism were mainly enriched in N-glycan biosynthesis, glycolysis and gluconeogenesis, starch and sucrose metabolism, and the down-regulated genes associated with glycometabolism were mainly enriched in fructose and mannose metabolism. In eukaryotes, the attachment and subsequent modification of N-glycans affect the folding of glycoproteins and regulate their secretion [28, 29]. *MOGS* and *GII alpha* were assigned to N-glycan biosynthesis, and their relative expression levels were up-regulated in response to DNJ (Fig 7). MOGS (also known as glucosidase I) is expressed in the endoplasmic reticulum and is involved in trimming N-glycans; it was the first enzyme to be identified in the pathway for processing N-linked oligosaccharides [30]. GII alpha belongs to glycoside hydrolase family 31 (GH31), which has similar functions as MOGS and is involved in trimming N-glycans [31]. As a recognized inhibitor of α-glucosidase, DNJ also inhibits MOGS and GII alpha activities [32–34]. However, the relative expression levels of the two genes showed an increasing trend in the 2% DNJ-treated group compared with the control. We speculated that the eri-silkworm might a produces stress response when glycosidase activity was inhibited by DNJ.*UGT* was enriched in the starch and sucrose metabolism pathway, and its expression was up-regulated in the 2% DNJ-treated group. UGTs are membrane-bound proteins that are mainly located on the luminal side of the endoplasmic reticulum (ER) in animals [35]. UGTs catalyse the conjugation of a range of diverse small lipophilic compounds with sugars to produce glycosides, playing an important role in the detoxification of xenobiotics and the regulation of endobiotics in insects [35–38]. Thus, UGT might be involved in the detoxification of DNJ and delaying the toxic effect.

### Lipid metabolism

Lipids are one of the three nutrients involved in energy storage, participate in cell membrane structure, hormone synthesis and vitamin storage. Lipid metabolism in insects is similar to mammals, comprising lipid absorption, transport, storage, and mobilization processes [39]. An insect that consumes a high sugar or high fat food for a long period will display disturbances in fat metabolism and lipotoxicity in organs [40]. DNJ has been shown to modulate lipid metabolism and prevent hyperlipidaemia [9,19,41,42]. In the present study, most of the genes involved in lipid metabolism were up-regulated after treatment with DNJ. These genes were related to fatty acid biosynthetic process, sphingolipid, glycerophospholipid and glycerolipid metabolism. According to the KEGG analysis, DGK and AR were enriched in glycerolipid metabolism. Diacylglycerol kinase isoforms regulate signal transduction and lipid metabolism and have divergent functional roles in distinct tissues [43]. AR is an NADPH-dependent reductase that is the first rate-limiting enzyme of the polyol pathway in glucose metabolism and is implicated in the pathogenesis of secondary diabetic complications [44]. In the last few decades, this enzyme has been used as a target to prevent cellular inflammatory events [45]. Based on the transcriptome sequencing and RT-qPCR results, *DGK* was up-regulated and *AR* was down-regulated in the 2% DNJ-treated group compared with the control. We speculated that DNJ modulated lipid metabolism by influencing the expression levels of *AR* and *DGK* in eri-silkworm. GABAT is a dimeric homopolymer that catalyses the first step in the conversion of the central inhibitory neurotransmitter gamma-amino butyric acid (GABA) to succinic acid [46]. As shown in our previous study, succinate levels were reduced in the haemolymph of fourth-instar eri-silkworm in response to the DNJ treatment [18]. Thus, DNJ inhibited the expression of the *GABAT* gene to influence succinate production.

### Energy metabolism

Energy metabolism is an important metabolic pathway in organisms and is the foundation of biological growth, development and life. In the present study, we focused on the genes related to oxidative phosphorylation, ATP binding, carbohydrate digestion and absorption pathway. Eighty-one DEGs related to energy metabolism were identified from transcriptome database. Oxidative phosphorylation is the metabolic pathway cells use enzymes to oxidize nutrients, thereby releasing the energy used to regenerate ATP [47]. In our previous study, DNJ had a significant effect on the TCA cycle in eri-silkworm. For example, DNJ reduced the fumarate and succinate levels in the metabolic pathway [18]. Most genes related to the oxidative phosphorylation pathway were down-regulated, indicating that DNJ might inhibit gene expression by modulating the TCA cycle. MRP49, ROS, and MRP1A are mainly related to the ATP binding pathway. The multidrug resistance protein is an ATP-binding cassette (ABC) transporter that is divided into eight subfamilies (from ABC-A to ABC-H) and transports a series of substrates across cellular membranes [48]. MDR1A (also known as p-glycoprotein) is a transport protein with a wide substrate specificity that belongs to the ATP-binding cassette protein family [49]. It plays an important role in drug excretion and is located in the apical membrane of cells in the intestine, kidney and liver to protect tissues from toxic xenobiotics and endogenous metabolites. In addition, it can affect the uptake and distribution of many clinically important drugs [50]. MRP49 and MRP1A belong to the ABC family; therefore, we postulated that these two genes were involved in protecting the eri-silkworm haemolymph from DNJ toxicity.

## Conclusions

In the present study, transcriptome sequencing was employed in eri-silkworm haemolymph for the first time to investigate the changes in genes related to glycometabolism, lipid metabolism and energy metabolism after the insects were fed DNJ. Our comprehensive analysis revealed effects on the three main metabolic pathways at the transcriptional level. Eight hundred sixty-five DEGs were identified, of which 138 DEGs were involved in energy metabolism, glycometabolism and lipid metabolism. Based on these results, DNJ influences gene expression levels to modulate glycometabolism, lipid metabolism and energy metabolism. These findings lay the foundation for obtaining a better understanding of the toxic effects of DNJ on eri-silkworm as a model and provide a reference for the exploitation of new drugs for humans.

## Supporting information

S1 Table Primers used for RT-qPCR to validate DEGs. (DOCX)

S2 Table Summary of the sequence assembly obtained after Illumina sequencing. (DOCX)

S3 Table Length frequency distribution of transcripts and unigenes. (DOCX)

S4 Table Identification of genes related to glycometabolism, lipid metabolism and energy metabolism. (DOCX)
