# Supplementary material for: Comparative transcriptome analysis reveals significant metabolic alterations in eri-silkworm (Samia cynthia ricini) haemolymph in response to 1-deoxynojirimycin
Source: PLoS One. 2018 Jan 11;13(1):e0191080. doi: 10.1371/journal.pone.0191080 (PMC5764371; doi:10.1371/journal.pone.0191080)
Supplement: S1 Table — (DOCX) [file pone.0191080.s001.docx]

**S1 Table. Primers used for RT-qPCR to validate DEGs.**

| **Unigenes ID** | **Best match** | **Primer sequences (5’ to 3’)** | **Length (bp)** |
| --- | --- | --- | --- |
| c29272_g1 | uncharacterized family 31 glucosidase KIAA1161 isoform X1 (*Bombyx mori*) | F: TCGGGCTGATGTTTCTCGC | 199 |
|  |  | R: AACGCCTTCACTTTTCCTTGC |  |
| c28736_g1 | chymotrypsin inhibitor CI-8A (*Bombyx mori*） | F:GAGTGGCTCTGTGGTAGTGTCC | 170 |
|  |  | R:TGAGATAAGCGAACGGGTGG |  |
| c29737_g1 | mannosyl-oligosaccharide glucosidase (*Bombyx mori*） | F:GATTTTGATAGAGACCTTAGCCCAT | 236 |
|  |  | R:GCACGATTAGTCCGCCAGC |  |
| c8813_g1 | uncharacterized protein LOC101744771 (*Bombyx mori*） | F:CTGTCAGGCAAGAAGGAATCG | 161 |
|  |  | R:CCAGAGTCGGTCGTGGTAGTAAG |  |
| c30429_g1 | glucosidase 2 subunit beta-like (*Bombyx mori*） | F:GGTAGGGCTGGGTAATTGGG | 169 |
|  |  | R:GCGGAAAGGTTCTGTGACTGAC |  |
| c38491_g1 | probable cyclin-dependent serine/threonine-protein kinase isoform X2 (*Bombyx mori*) | F:GTCGCCGTTCTGGTCTTGG | 141 |
|  |  | R:CTTCGTGGTCGCATTGTTCC |  |
| c38144_g1 | protease inhibitor-like protein (*Antheraea mylitta*) | F:TACTGCCCACAACAAGCGG | 146 |
|  |  | R:GACAGTTTGACGCACTTTCCG |  |
| c25580_g1 | N-glycosylase/DNA lyase-like(*Plutella xylostella*) | F:CGGCACAGAACTACCTCCCAC | 191 |
|  |  | R:GGTTTAGCGTTATCATTATCATCGG |  |
| c23049_g1 | glucosidase II alpha-subunit (*Spodoptera frugiperda*) | F:GCGGAAGAACGGCTGGTAA | 276 |
|  |  | R:ACGCACGAGGGTATGCTGAA |  |
| c28437_g7 | trypsin, partial (*Manduca sexta*) | F:AGAGCAGACACCGACGACAA | 121 |
|  |  | R:TGACACTATCACCGCCAACA |  |
| c38104_g1 | Kruppel homolog 1 (*Bombyx mori*) | F:CTGTCGGTTGAGCGAGTGTCT R:TGATGATTTAGTGGCGAGGTGT | 105 |
| c29805_g3 | multidrug resistance protein 1A (*Bombyx mori*) | F:ATTGATGGTCTGGTCGGGTAT  R: AGAGGCACTTCTTATGGGGG | 213 |
| c29290_g1 | multidrug resistance protein homolog 49-like(*Bombyx mori*) | F:TTCTCCGTTCACTCCCCAG  R:CGCACACAGTAACAACATTCG | 127 |
| c29308_g1 | proto-oncogene tyrosine-protein kinase ROS isoform X1 (*Bombyx mori*) | F:AACCTTCGCATACAGTCCCC  R:GCCACCTCCGCATTACCTT | 212 |
| c28893_g1 | UDP-glucosyltransferase precursor (*Bombyx mori*) | F: CGGCGGTTGTCTTCCTCTC  R: TCCATTTATTTGGGTGGGTAGT | 256 |
| c46112_g1  c26875_g1  c26651_g1 | 4-aminobutyrate aminotransferase, mitochondrial (*Bombyx mori*)  eye-specific diacylglycerol kinase isoform X3 (*Bombyx mori*)  aldose reductase-like (*Bombyx mori*) | F: CAATCTGCCGAGGGAAACA  R: TTGATGCGGATGGTAATGAGT  F: CAACCTGAAACGACTGCCTCT  R: GATTCGGGAACATCCAACG  F: AAGGTCTTGAAGCGTAGTTTTGA  R: AGGAAGTTGGCGAGGCATT | 160  140  143 |
|  | β-actin | F:GGGCCGGACTCGTCATATT | 153 |
|  |  | R:ATCACAGCCCTCGCTCGCTCCAT |  |
