# Supplementary material for: Comparative transcriptome analysis reveals significant metabolic alterations in eri-silkworm (Samia cynthia ricini) haemolymph in response to 1-deoxynojirimycin
Source: PLoS One. 2018 Jan 11;13(1):e0191080. doi: 10.1371/journal.pone.0191080 (PMC5764371; doi:10.1371/journal.pone.0191080)
Supplement: S2 Table — (DOCX) [file pone.0191080.s002.docx]

**S2 Table. Summary of the sequence assembly obtained after Illumina sequencing**

| **Groups** | **Raw Reads** | **Clean Reads** | **Clean Bases** | **Error (%)** | **Q20^a^ (%)** | **Q30^b^ (%)** | **GC Content (%)** |
| --- | --- | --- | --- | --- | --- | --- | --- |
| Con-1 | 46,811,398 | 45,386,222 | 6.81G | 0.02 | 96.08 | 90.88 | 45.8 |
| Con-2 | 57,836,584 | 56,099,312 | 8.41G | 0.02 | 96.14 | 91.02 | 45.72 |
| Con-3 | 48,797,240 | 47,216,414 | 7.08G | 0.02 | 96.04 | 90.83 | 46.42 |
| 2% DNJ-1 | 44,655,898 | 43,437,882 | 6.49G | 0.02 | 96.42 | 91.56 | 45.23 |
| 2% DNJ-2 | 42,290,810 | 41,263,002 | 6.17G | 0.02 | 96.65 | 91.91 | 46.43 |
| 2% DNJ-3 | 42,821,388 | 41,636,180 | 6.22G | 0.02 | 96.33 | 91.34 | 46.65 |

^a^Q20: percentage of baseswith a Phred value of at least 20.

^b^Q30: percentage of bases with a Phred value of at least 30.
