# Supplementary material for: Comparative transcriptome analysis reveals significant metabolic alterations in eri-silkworm (Samia cynthia ricini) haemolymph in response to 1-deoxynojirimycin
Source: PLoS One. 2018 Jan 11;13(1):e0191080. doi: 10.1371/journal.pone.0191080 (PMC5764371; doi:10.1371/journal.pone.0191080)
Supplement: S3 Table — (DOCX) [file pone.0191080.s003.docx]

**S3 Table. Length frequency distribution of transcripts and unigenes**

| **Transcript length interval** | **200-500bp** | **500-1kbp** | **1k-2kbp** | **>2kbp** | **Total** |
| --- | --- | --- | --- | --- | --- |
| **Number of transcripts** | 55,807 | 12,956 | 8,047 | 9,509 | 86,319 |
| **Number of unigenes** | 51,652 | 10,508 | 5,573 | 5,563 | 73,296 |
