# Supplementary material for: Comparative transcriptome analysis reveals significant metabolic alterations in eri-silkworm (Samia cynthia ricini) haemolymph in response to 1-deoxynojirimycin
Source: PLoS One. 2018 Jan 11;13(1):e0191080. doi: 10.1371/journal.pone.0191080 (PMC5764371; doi:10.1371/journal.pone.0191080)
Supplement: S4 Table — (DOCX) [file pone.0191080.s004.docx]

**S4 Table. Identification of genes related to glycometabolism, lipid metabolism and energy metabolism**

| **Gene ID** | **Nr annotation** | **2%-DNJ**  **FPKM** | **H_2_O**  **FPKM** | **2%-DNJ vs. H_2_O ratio** | ***p*-value** |
| --- | --- | --- | --- | --- | --- |
| **Glycometabolism** | | | | | |
| c10515_g1 | phosphoenolpyruvate carboxykinase, putative (*Eimeria tenella*) | 1.18 | 0.00 | NA^a^ | 9.04E-05 |
| c24239_g1 | uncharacterized protein LOC105367142, partial (*Ceratosolen solmsi marchali*) | 0.67 | 0.00 | NA | 5.31E-07 |
| c38751_g1 | fibrinogen beta chain precursor (*Rattus norvegicus*) | 0.00 | 0.80 | 0.00 | 5.63E-11 |
| c27124_g2 | triosephosphate isomerase isoform X1 (*Sus scrofa*) | 0.76 | 0.00 | NA | 3.68E-07 |
| c24593_g2 | poly (ADP-ribose) polymerase 6 isoform X10 (*Sus scrofa*) | 0.96 | 0.16 | 5.88 | 1.66E-09 |
| c29813_g1 | light-harvesting complex protein LHCG11 (*Bigelowiella natans*) | 10.83 | 0.00 | NA | 6.02E-20 |
| c4751_g1 | phosphoglucomutase-1 (*Bos taurus*) | 0.79 | 0.00 | NA | 9.37E-17 |
| c24226_g1 | phosphoglycerate mutase 2 (*Sus scrofa*) | 3.27 | 0.00 | NA | 2.90E-19 |
| c25080_g1 | fructose-bisphosphate aldolase A isoform X2 (*Odobenus rosmarus divergens*) | 7.50 | 0.07 | 102.32 | 5.61E-39 |
| c68361_g1 | glucose-6-phosphate isomerase (*Sus scrofa*) | 0.52 | 0.00 | NA | 6.63E-08 |
| c20410_g1 | glyceraldehyde-3-phosphate dehydrogenase (*Sus scrofa*) | 16.50 | 0.23 | 72.78 | 2.68E-72 |
| c13206_g1 | beta-enolase isoform X1 (*Sus scrofa*) | 5.49 | 0.00 | NA | 1.19E-34 |
| c17737_g1 | mitochondrial phosphoenolpyruvate carboxykinase isoform 1 (*Bombyx mori*) | 16.79 | 25.02 | 0.67 | 1.63E-12 |
| c27124_g1 | triosephosphate isomerase (*Mus musculus*) | 0.00 | 0.62 | 0.00 | 0.00042072 |
| c22099_g1 | alpha-1-antiproteinase precursor (*Rattus norvegicus*) | 0.00 | 2.03 | 0.00 | 1.45E-18 |
| c8826_g1 | fructose-bisphosphate aldolase C (*Mus musculus*) | 0.00 | 0.74 | 0.00 | 1.50E-05 |
| c17731_g1 | alpha-(1,6)-fucosyltransferase-like (*Bombyx mori*) | 9.45 | 6.68 | 1.41 | 0.00026536 |
| c29737_g1 | mannosyl-oligosaccharide glucosidase (*Bombyx mori*) | 6.48 | 4.53 | 1.43 | 0.00010028 |
| c10794_g1 | alpha-mannosidase 2 (*Bombyx mori*) | 9.92 | 6.24 | 1.59 | 4.05E-08 |
| c30351_g1 | dolichyl-diphosphooligosaccharide--protein glycosyltransferase subunit STT3A (*Bombyx mori*) | 104.13 | 75.12 | 1.39 | 7.19E-06 |
| c27428_g1 | glyceraldehyde-3-phosphate dehydrogenase (*Littorina littorea*) | 0.64 | 0.00 | NA | 1.23E-06 |
| c25014_g1 | acetyl-coenzyme A synthetase, chloroplastic/glyoxysomal-like, partial (*Ceratosolen solmsi marchali*) | 0.60 | 0.00 | NA | 3.39E-07 |
| c37694_g1 | aldehyde dehydrogenase, mitochondrial (*Plutella xylostella*) | 237.60 | 183.96 | 1.29 | 0.0003048 |
| c5022_g1 | dolichyl-diphosphooligosaccharide--protein glycosyltransferase subunit STT3B isoform X2 (*Bombyx mori*) | 34.61 | 24.69 | 1.40 | 5.07E-06 |
| c27923_g1 | alpha-(1,3)-fucosyltransferase C-like (*Bombyx mori*) | 20.26 | 26.90 | 0.75 | 1.08E-06 |
| c28238_g1 | alpha 1,2-mannosidase (*Spodoptera frugiperda*) | 27.31 | 17.84 | 1.53 | 1.52E-08 |
| c26651_g1 | aldose reductase-like (*Bombyx mori*) | 14.36 | 33.90 | 0.42 | 4.59E-11 |
| c12908_g1 | mitochondrial enolase superfamily member 1-like (*Bombyx mori*) | 35.31 | 45.51 | 0.78 | 3.09E-06 |
| c16928_g1 | sorbitol dehydrogenase (*Bombyx mori*) | 21.32 | 34.23 | 0.62 | 5.25E-11 |
| c30387_g1 | trehalose-6-phosphate synthase (*Spodoptera litura*) | 13.48 | 16.86 | 0.80 | 1.86E-05 |
| c68982_g1 | UDP-glycosyltransferase UGT44A2 (*Helicoverpa armigera*) | 2.10 | 1.12 | 1.88 | 0.00066021 |
| c28893_g1 | UDP-glucosyltransferase precursor (*Bombyx mori*) | 13.85 | 6.68 | 2.07 | 5.99E-14 |
| c28920_g1 | glycogen phosphorylase, muscle form isoformX1 (*Sus scrofa*) | 1.93 | 0.00 | NA | 5.04E-48 |
| c23311_g1 | UDP-glycosyltransferase UGT340C1 precursor (*Bombyx mori*) | 19.30 | 10.97 | 1.76 | 2.10E-11 |
| c53175_g1 | NADP-dependent malic enzyme isoform X2 (*Plutella xylostella*) | 302.77 | 446.32 | 0.68 | 6.30E-09 |
| **S4 Table** (continued) | | | | | |
| **Gene ID** | **Nr annotation** | **2%-DNJ**  **FPKM** | **H_2_O**  **FPKM** | **2%-DNJ vs. H_2_O ratio** | ***p*-value** |
| c29784_g1 | acetyl-CoA carboxylase isoform X1 (*Bombyx mori*) | 6.35 | 4.53 | 1.40 | 6.38E-05 |
| c23049_g1 | glucosidase II alpha-subunit (*Spodoptera frugiperda*) | 105.79 | 59.97 | 1.76 | 2.24E-19 |
|  |  |  |  |  |  |
| **Energy metabolism** | | | | | |
| c57425_g1 | cytochrome oxidase subunit I, partial (mitochondrion) (*Rhabdomys dilectus*) | 0.00 | 2.93 | 0.00 | 7.94E-08 |
| c26068_g1 | COII (*Sus scrofa*) | 7.52 | 0.09 | 80.54 | 9.62E-08 |
| c25970_g1 | ATP synthase F0 subunit 6 (*Sus scrofa*) | 7.98 | 0.07 | 114.00 | 1.34E-39 |
| c40402_g1 | cytochrome oxidase subunit III (*Manis tetradactyla*) | 0.00 | 6.18 | 0.00 | 4.73E-26 |
| c26068_g2 | cytochrome c oxidase subunit II, partial (mitochondrion) (*Capra hircus*) | 9.95 | 0.61 | 16.40 | 0.00035651 |
| c25137_g1 | NADH dehydrogenase subunit 5 (mitochondrion) (*Sus scrofa*) | 0.72 | 0.00 | NA | 0.00049689 |
| c56772_g1 | cytochrome c oxidase subunit II (mitochondrion) (*Mus musculus domesticus*) | 0.04 | 4.10 | 0.01 | 7.25E-12 |
| c61881_g1 | cytochrome oxidase subunit I, partial (mitochondrion) (*Rattus tanezumi*) | 0.00 | 10.68 | 0.00 | 7.16E-07 |
| c26731_g1 | cytochrome c oxidase subunit III (*Sus scrofa*) | 6.37 | 0.09 | 68.25 | 1.83E-29 |
| c27070_g2 | NADH dehydrogenase subunit 1, partial (mitochondrion) (*Rattus norvegicus*) | 0.00 | 1.93 | 0.00 | 2.06E-13 |
| c27070_g1 | NADH dehydrogenase subunit 1 (*Sus scrofa*) | 2.03 | 0.00 | NA | 2.40E-07 |
| c21936_g1 | beta-subunit (AA 1-312) (*Homo sapiens*) | 0.00 | 0.48 | 0.00 | 0.0004067 |
| c28157_g1 | cytochrome c oxidase subunit I (*Sus scrofa*) | 5.05 | 0.01 | 378.75 | 5.21E-63 |
| c28157_g2 | cytochrome c oxidase subunit I (*Mus musculus musculus*) | 0.02 | 10.01 | 0.00 | 1.97E-57 |
| c28720_g1 | NADH dehydrogenase subunit 4 (*Sus scrofa*) | 1.12 | 0.03 | 33.50 | 8.01E-13 |
| c26516_g1 | probable NADH dehydrogenase (ubiquinone) 1 alpha subcomplex subunit 12 (*Bombyx mori*) | 109.92 | 143.58 | 0.77 | 0.00020207 |
| c26023_g2 | NADH dehydrogenase subunit 2 (*Rattus norvegicus*) | 0.26 | 1.13 | 0.23 | 7.80E-05 |
| c26023_g1 | NADH dehydrogenase subunit 2 (mitochondrion) (*Vulpes lagopus*) | 1.03 | 0.04 | 23.77 | 8.81E-07 |
| c17020_g2 | ATP synthase F0 subunit 6 (mitochondrion) (*Leopoldamys edwardsi*) | 0.00 | 3.03 | 0.00 | 1.05E-12 |
| c17020_g1 | ATP synthase F0 subunit 6 (mitochondrion) (*Mus spretus*) | 0.00 | 2.22 | 0.00 | 8.78E-08 |
| c26542_g2 | GM12097 (*Drosophila sechellia*) | 0.81 | 0.00 | NA | 0.00071922 |
| c68678_g1 | NADH dehydrogenase subunit 4 (*Rattus norvegicus*) | 0.00 | 0.81 | 0.00 | 1.42E-11 |
| c46151_g1 | H+ transporting ATP synthase subunit d (*Bombyx mori*) | 469.33 | 546.05 | 0.86 | 0.00056754 |
| c54004_g1 | sodium/potassium-transporting ATPase subunit beta-1 (*Rattus norvegicus*) | 0.00 | 0.46 | 0.00 | 2.65E-06 |
| c30404_g1 | hypothetical protein KGM_22373 (*Danaus plexippus*) | 36.49 | 45.49 | 0.80 | 3.27E-05 |
| c6036_g1 | sodium/potassium-transporting ATPase subunit alpha isoform X4 (*Bombyx mori*) | 36.57 | 25.78 | 1.42 | 6.33E-07 |
| c68292_g1 | myrosinase 1-like (*Bombyx mori*) | 19.33 | 12.31 | 1.57 | 8.11E-07 |
| c61757_g1 | glycosyl hydrolase family protein (*Naegleria gruberi*) | 0.43 | 0.00 | NA | 6.26E-05 |
| c29963_g1 | DNA mismatch repair protein Msh2 isoform X1 (*Bombyx mori*) | 9.02 | 11.51 | 0.78 | 0.00015374 |
| c13162_g1 | unnamed protein product (*Vitrella brassicaformis CCMP3155*) | 0.55 | 0.00 | NA | 0.00065713 |
| c28423_g2 | serine/threonine-protein kinase Genghis Khan (*Bombyx mori*) | 7.48 | 5.28 | 1.42 | 7.64E-06 |
| c27074_g1 | galactokinase-like isoform X1 (*Bombyx mori*) | 39.99 | 51.22 | 0.78 | 1.42E-05 |
| c29784_g1 | acetyl-CoA carboxylase isoform X1 (*Bombyx mori*) | 6.35 | 4.53 | 1.40 | 6.38E-05 |
| c9541_g1 | adenylate kinase isoenzyme 1 isoformX1 (*Sus scrofa*) | 1.05 | 0.00 | NA | 2.38E-06 |
| c23754_g1 | imilar to CG5304-PA (*Papilio xuthus*) | 15.26 | 19.15 | 0.80 | 0.00014014 |
| c7660_g1 | adenylate kinase 7-like (*Plutella xylostella*) | 11.62 | 15.19 | 0.77 | 7.76E-05 |
| c28582_g1 | ATP-binding cassette sub-family A member 3-like (*Plutella xylostella*) | 4.78 | 3.40 | 1.41 | 0.00010187 |
| **S4 Table** (Continued) | | | | | |
| **Gene ID** | **Nr annotation** | **2%-DNJ**  **FPKM** | **H_2_O**  **FPKM** | **2%-DNJ vs. H_2_O ratio** | ***p*-value** |
| c28625_g2 | SPARC-related modular calcium-binding protein 1 (*Bombyx mori*) | 11.80 | 7.46 | 1.58 | 1.17E-08 |
| c20784_g3 | kinesin-like protein unc-104 isoform X5 (*Bombyx mori*) | 5.14 | 3.34 | 1.54 | 1.24E-07 |
| c30328_g1 | dynein heavy chain, cytoplasmic (*Plutella xylostella*) | 10.92 | 8.00 | 1.36 | 1.51E-05 |
| c15776_g1 | S-adenosylmethionine synthetase (*Bombyx mori*) | 170.63 | 205.66 | 0.83 | 2.46E-05 |
| c29123_g2 | putative annexin IX-C (*Manduca sexta*) | 612.09 | 456.74 | 1.34 | 0.00066136 |
| c6996_g1 | ras-related and estrogen-regulated growth inhibitor (*Bombyx mori*) | 11.24 | 15.56 | 0.72 | 3.12E-05 |
| c14022_g1 | ATP-binding cassette transporter subfamily B isoform X1 (*Bombyx mori*) | 12.49 | 8.73 | 1.43 | 8.23E-06 |
| c25792_g1 | CTL-like protein 2 isoform X3 (*Plutella xylostella*) | 13.63 | 9.37 | 1.45 | 6.73E-06 |
| c45821_g1 | chaperonin subunit 6a zeta (*Bombyx mori*) | 119.78 | 141.80 | 0.84 | 0.00015176 |
| c29510_g1 | GTP-binding protein sar1 (*Papilio xuthus*) | 76.87 | 50.14 | 1.53 | 2.07E-08 |
| c29603_g1 | alanine--glyoxylate aminotransferase 2, mitochondrial-like (*Bombyx mori*) | 16.51 | 24.36 | 0.68 | 4.47E-10 |
| c21936_g1 | beta-subunit (AA 1-312) (*Homo sapiens*) | 0.00 | 0.48 | 0.00 | 0.0004067 |
| c26979_g1 | hypothetical protein KGM_15700 (*Danaus plexippus*) | 5.72 | 3.88 | 1.47 | 0.00027057 |
| c29864_g2 | conventional protein kinase C isoform X1 (*Bombyx mori*) | 16.94 | 12.43 | 1.36 | 1.37E-05 |
| c28390_g2 | proton-coupled amino acid transporter 4 (*Bombyx mori*) | 16.62 | 8.76 | 1.90 | 4.53E-21 |
| c10515_g1 | phosphoenolpyruvate carboxykinase, putative (*Eimeria tenella*) | 1.18 | 0.00 | NA | 9.04E-05 |
| c15815_g1 | integral membrane protein 2B (*Mus musculus*) | 0.00 | 0.49 | 0.00 | 3.18E-05 |
| c15978_g1 | tyrosine kinase receptor Cad96Ca (*Bombyx mori*) | 6.78 | 4.51 | 1.50 | 0.00019706 |
| c68430_g1 | UPF0489 protein C5orf22 homolog isoform X1 (*Bombyx mori*) | 11.21 | 15.07 | 0.74 | 7.63E-05 |
| c27150_g1 | triple functional domain protein (*Plutella xylostella*) | 25.64 | 17.78 | 1.44 | 5.17E-07 |
| c28519_g1 | ribonuclease L inhibitor homolog isoform X1 (*Bombyx mori*) | 143.20 | 178.01 | 0.80 | 2.81E-06 |
| c26891_g1 | serine/threonine-protein kinase RIO2 (*Bombyx mori*) | 32.73 | 38.99 | 0.84 | 0.00050828 |
| c29290_g1 | multidrug resistance protein homolog 49-like (*Bombyx mori*) | 46.33 | 24.00 | 1.93 | 6.46E-06 |
| c29308_g1 | proto-oncogene tyrosine-protein kinase ROS isoform X1 (*Bombyx mori*) | 2.12 | 0.69 | 3.07 | 8.44E-23 |
| c68114_g1 | putative serine/threonine protein kinase (*Danaus plexippus*) | 305.74 | 380.94 | 0.80 | 1.24E-06 |
| c17813_g1 | Endoprotease FURIN (*Spodoptera frugiperda*) | 7.05 | 5.12 | 1.38 | 0.0001323 |
| c29354_g1 | ATP-binding cassette sub-family G member 1, partial (*Bombyx mori*) | 7.19 | 9.05 | 0.79 | 0.00020601 |
| c19185_g1 | Eph receptor (*Manduca sexta*) | 8.77 | 10.89 | 0.81 | 0.00010156 |
| c22028_g2 | cyclin dependent kinase 4 (*Bombyx mori*) | 5.89 | 7.72 | 0.76 | 0.00061047 |
| c28761_g1 | myosin vi (*Danaus plexippus*) | 9.26 | 6.46 | 1.43 | 5.06E-06 |
| c27785_g1 | uncharacterized protein LOC101742402 isoform X1 (*Bombyx mori*) | 20.33 | 25.30 | 0.80 | 0.0002517 |
| c423_g1 | ATP-binding cassette sub-family G member 1 (*Bombyx mori*) | 4.49 | 2.97 | 1.51 | 0.00057982 |
| c29123_g3 | putative annexin IX-B (*Manduca sexta*) | 1172.83 | 1468.33 | 0.80 | 0.0006136 |
| c30234_g1 | unconventional myosin-XVIIIa, partial (*Bombyx mori*) | 21.39 | 15.31 | 1.40 | 2.65E-11 |
| c21627_g1 | PKG-Ib (*Danaus plexippus*) | 25.91 | 19.07 | 1.36 | 2.51E-05 |
| c68110_g1 | ABCC4-like protein (*Spodoptera litura*) | 18.42 | 12.34 | 1.49 | 3.11E-08 |
| c30216_g1 | myosin-4 (*Sus scrofa*) | 4.76 | 0.00 | NA | 6.83E-119 |
| c29290_g2 | multidrug resistance protein homolog 49-like (*Bombyx mori*) | 35.94 | 19.83 | 1.81 | 0.00072526 |
| c24940_g1 | multidrug resistance-associated protein 7 (*Bombyx mori*) | 7.25 | 5.31 | 1.37 | 0.00020997 |
| c29189_g1 | serine/threonine-protein kinase/endoribonuclease IRE1-like (*Bombyx mori*) | 7.35 | 5.30 | 1.39 | 0.00065565 |
| c25993_g1 | apolipoprotein E precursor (*Rattus norvegicus*) | 0.00 | 5.10 | 0.00 | 5.89E-43 |
| **S4 Table** (Continued) | | | | | |
| **Gene ID** | **Nr annotation** | **2%-DNJ**  **FPKM** | **H_2_O**  **FPKM** | **2%-DNJ vs. H_2_O ratio** | ***p*-value** |
| c22311_g1 | DEAD box polypeptide 5 isoform 1 (*Bombyx mori*) | 839.06 | 963.51 | 0.87 | 0.00063226 |
| c30039_g1 | myosin heavy chain, non-muscle-like (*Plutella xylostella*) | 132.67 | 87.60 | 1.51 | 7.45E-06 |
| c30257_g2 | NFX1-type zinc finger-containing protein 1-like isoform X1 (*Plutella xylostella*) | 53.30 | 64.83 | 0.82 | 2.45E-06 |
| c29805_g3 | multidrug resistance protein 1A (*Bombyx mori*) | 2.52 | 5.80 | 0.43 | 2.53E-10 |
|  |  |  |  |  |  |
| **Lipid metabolism** | | | | | |
| c71439_g1 | apolipoprotein C-III precursor (*Rattus norvegicus*) | 0.00 | 3.21 | 0.00 | 2.36E-08 |
| c46112_g1 | 4-aminobutyrate aminotransferase, mitochondrial (*Bombyx mori*) | 1.27 | 2.25 | 0.57 | 0.00017953 |
| c29784_g1 | acetyl-CoA carboxylase isoform X1 (*Bombyx mori*) | 6.35 | 4.53 | 1.40 | 6.38E-05 |
| c45460_g1 | estradiol 17-beta-dehydrogenase 11-like (*Bombyx mori*) | 98.59 | 76.20 | 1.29 | 0.00057593 |
| c23755_g1 | prominin-like protein (*Plutella xylostella*) | 13.65 | 8.28 | 1.65 | 1.90E-05 |
| c30930_g1 | fatty acid hydroxylase domain-containing protein 2 (*Geospiza fortis*) | 0.44 | 0.00 | NA | 0.00050742 |
| c8049_g1 | diacylglycerol O-acyltransferase 1 isoform X1 (*Bombyx mori*) | 16.45 | 12.20 | 1.35 | 0.00045424 |
| c68195_g1 | Chain A, Liver Fatty Acid Binding Protein-Oleate Complex (*Microtus ochrogaster*) | 0.00 | 1.72 | 0.00 | 0.00015766 |
| c29136_g1 | ceramide synthase 5-like (*Bombyx mori*) | 13.24 | 8.38 | 1.58 | 2.70E-06 |
| c28480_g3 | sphingolipid delta(4)-desaturase DES1 (*Bombyx mori*) | 24.81 | 17.05 | 1.46 | 0.000000504 |
| c7889_g1 | neutral ceramidase (*Bombyx mori*) | 39.27 | 28.10 | 1.40 | 8.74E-06 |
| c27491_g1 | sphingomyelin phosphodiesterase isoform X1 (*Bombyx mori*) | 33.83 | 21.35 | 1.58 | 3.01E-10 |
| c27512_g1 | diacylglycerol kinase 1 (*Bombyx mori*) | 9.16 | 6.37 | 1.44 | 0.0000663 |
| c27774_g1 | fatty alcohol acetyltransferase (*Agrotis segetum*) | 55.02 | 40.98 | 1.34 | 7.15E-05 |
| c10505_g1 | glycerol-3-phosphate dehydrogenase (NAD+), cytoplasmic (*Sus scrofa*) | 0.66 | 0.00 | NA | 2.14E-04 |
| c22352_g1 | ethanolaminephosphotransferase 1-like (*Bombyx mori*) | 48.08 | 62.05 | 0.77 | 0.000000191 |
| c37733_g1 | glycerol-3-phosphate dehydrogenase isoform 2 (*Bombyx mori*) | 22.84 | 13.61 | 1.68 | 1.24E-12 |
| c26875_g1 | eye-specific diacylglycerol kinase isoform X3 (*Bombyx mori*) | 3.19 | 1.82 | 1.75 | 1.53E-05 |
| c37694_g1 | aldehyde dehydrogenase, mitochondrial (*Plutella xylostella*) | 237.60 | 183.96 | 1.29 | 0.0003048 |
| c26651_g1 | aldose reductase-like (*Bombyx mori*) | 14.36 | 33.90 | 0.42 | 4.59E-11 |

^a^NA: not applicable.
